# Bovine Lactoferrin-Loaded Plasmonic Magnetoliposomes for Antifungal Therapeutic Applications

**DOI:** 10.3390/pharmaceutics15082162

**Published:** 2023-08-19

**Authors:** Mélanie Pereira, Ana Rita O. Rodrigues, Leslie Amaral, Manuela Côrte-Real, Cátia Santos-Pereira, Elisabete M. S. Castanheira

**Affiliations:** 1 Physics Centre of Minho and Porto Universities (CF-UM-UP), University of Minho, Campus de Gualtar, 4710-057 Braga, Portugal; 2 LaPMET (Laboratory of Physics for Materials and Emergent Technologies), Associate Laboratory, 4710-057 Braga, Portugal; 3 Centre of Molecular and Environmental Biology (CBMA), Department of Biology, University of Minho, 4710-057 Braga, Portugal; 4 CEB—Centre of Biological Engineering, University of Minho, Campus de Gualtar, 4710-057 Braga, Portugal

**Keywords:** bovine lactoferrin, plasmonic magnetoliposomes, dual hyperthermia, antifungal activity, cytotoxicity

## Abstract

Bovine lactoferrin (bLf) is a milk-derived protein that exhibits potent broad-spectrum antifungal activity against multiple fungi. bLf is susceptible to degradation, while some of its properties depend on the tertiary structure. So, the encapsulation of bLf in stimuli-responsive therapeutic formulations provides an added value to enhance its biological activities. Plasmonic magnetoliposomes (PMLs) arise as promising nanocarriers for dual hyperthermia (magneto-photothermia) and local chemotherapy, since the combination of magnetic and gold nanoparticles (NPs) in a single nanosystem (multifunctional liposomes) enables the targeting and controlled release of loaded drugs. In this work, plasmonic magnetoliposomes (PMLs) containing manganese ferrite nanoparticles (28 nm size) and gold nanoparticles (5–7.5 nm size), functionalized with 11-mercaptoundecanoic acid or octadecanethiol, were prepared and loaded with bLf. The NPs’ optical, magnetic and structural properties were measured via UV/vis/NIR absorption spectroscopy, SQUID and TEM, respectively. The Specific Absorption Rate (SAR) was calculated to assess the capabilities for magnetic and photothermal hyperthermia. Finally, the antifungal potential of bLf-loaded PMLs and their mechanism of internalization were assessed in *Saccharomyces cerevisiae* by counting the colony forming units and using fluorescence microscopy. The results demonstrate that PMLs are mainly internalized through an energy- and temperature-dependent endocytic process, though the contribution of a diffusion component cannot be discarded. Most notably, only bLf-loaded plasmonic magnetoliposomes display cytotoxicity with an efficiency similar to free bLf, attesting their promising potential for bLf delivery in the context of antifungal therapeutic interventions.

## 1. Introduction

Fungal diseases are the cause of about 13 million infections and more than 1.5 million deaths per year worldwide [1,2]. Fungi can cause superficial or cutaneous disease in deep tissue, as well as life-threatening systemic infections. Most clinically relevant fungal infections affect patients with impaired immune function, such as patients with cancer or acquired immunodeficiency syndrome (AIDS) [1]. Infections caused by *Candida albicans*, *Aspergillus fumigatus* and *Cryptococcus neoformans* are the most common. However, there is an increasing problem associated with the augmented frequency of infections caused by other fungi, which are more difficult to diagnose and more resistant to the currently used antifungal agents [1]. Conventional therapies for treating fungal infections are usually administered by topical application or through oral delivery, which were demonstrated to be promising for different therapeutic applications [3,4,5]. However, these methods present several drawbacks. In the case of topical application, retention is inefficient in the upper layers of the skin, while in oral administration, systemic toxicity is commonly an issue [6]. The encapsulation of antifungal compounds into nanoparticles has been emerging as a promising strategy to overcome the problems of conventional therapies by reducing side effects, enhancing drug solubility, maximizing antifungal activity and bioavailability and possibly extending antifungal drug action, as reviewed by others [7,8,9]. On the other hand, the number of multi-resistant fungi has been increasing over the past few decades, which is a serious concern in clinical practice and reinforces the need for alternative antifungals [10]. Thus, finding new antifungals and innovative antifungal strategies is of the utmost importance to counteract the worldwide burden of fungal infections.

Lactoferrin (Lf) is an 80 kDa iron-binding glycoprotein of the transferrin family produced by mucosal epithelial cells or neutrophils during inflammation processes. It is present in several biological fluids, such as tears, saliva, sweat and, in greater abundance, in milk [11]. Lf has been described as a multifunctional protein exhibiting antibacterial, anti-viral, anti-inflammatory, antifungal, anticancer and immunomodulatory activities [12,13,14,15,16]. Among those, its antifungal activity stands out [5,13], as the numerous reports available in the literature demonstrate its potent action against several different fungi, suggesting that Lf as a broad-spectrum natural antifungal agent. Indeed, Lf was shown to display fungicidal activity against several *Candida* species [17,18], dermatophytic fungi, such as *Trichophyton mentagrophytes* and *T. rubrum* [19], and fungi isolated from plants and soils, including *Aspergillus niger*, *Trichoderma viride*, *Sclerotinia sclerotiorum*, *Sclerotium rolfsii*, *Rhizoctonia solani*, *Phoma exigua* [19] and *Botrytis cinerea* [20]. More recently, a study aiming to evaluate Lf antifungal activity against a wide range of yeasts and molds, including clinical, veterinary and environmental isolates, concluded that it was toxic against all the tested strains (46 in total) from the genus *Candida*, *Saccharomyces*, *Cryptococcus*, *Meyerozyma*, *Kluyveromyces*, *Pichia* and *Clavispora* [21]. As a result of this plethora of studies, pre-clinical and clinical studies have been published recently that demonstrate the excellent potential of Lf as an antifungal agent. Lf treatment promoted fungi clearance in most organs, decreased infection foci and downregulated virulence-associated genes in a mouse model of *C. albicans* systemic infection [22]. A promising strategy against vulvovaginal candidiasis (VVC) was reported in a VVC mouse model using a *Lactobacillus casei* strain secreting Lf [23]. A clinical trial in women with recurrent VVC infections using a similar strategy (a Lactobacilli mixture in combination with Lf but with an intake of clotrimazole at the beginning of the trial) concluded that the treatment significantly decreased candidiasis recurrence [5]. However, when Lf is orally administered, it was shown to undergo degradation in the gastrointestinal tract, generating Lf-derived peptides. Though this degradation may generate smaller peptides with antifungal activity [24], it may compromise some of the Lf properties and biological activities that depend on its tertiary structure [25,26]. Recently, different approaches have been developed to encapsulate Lf and help overcome these issues [27,28].

Nanoparticles (NPs), with sizes ranging from 1 to 200 nm, exhibit numerous applications in the biomedical field, such as for diagnosis through atomic imaging, for magnetic and photothermal therapy and targeted drug delivery, among others [29]. In the last few decades, liposomes have gained great importance in biomedicine. They are small spherical vesicles composed of at least one lipid bilayer and have been described as ideal systems for the encapsulation and delivery of bioactive compounds [30]. Magnetic nanoparticles with superparamagnetic behavior are advantageous for biomedical use, since their magnetic properties are only manifested in the presence of a magnetic field, making it possible to direct them to the target site and cause hyperthermia without causing cytotoxicity to healthy cells [31,32]. Plasmonic nanoparticles, which convert part of the absorbed energy into heat, can also be encapsulated into liposomes and be used for photothermal therapy (PTT). The Surface Plasmon Resonance (SPR) phenomenon of noble-metal-based nanoparticles makes it possible to generate heat through the absorption of energy from a laser. PTT enhances the action of chemical agents because it can: (i) accumulate more nanocarriers in the affected tissue, (ii) facilitate cell membrane permeability, (iii) increase the cytotoxic effect of the drug, and (iv) release the drug at the target site [33,34,35]. The conjugation of magnetic and plasmonic NPs into a single nanosystem (multifunctional liposomes) is of great interest in nanomedicine for dual hyperthermia (magneto-photothermal).

Hitherto, Lf nanocarriers have been commonly synthesized by methods, such as sol–oil or water-in-oil emulsions, or through thermal denaturation [36]. These nanosystems have numerous applications, including the transport and release of the protein to the target sites, diagnosis and treatment of cancers, treatment of infections and for use in the food industry [37]. While these formulations exhibit several advantages, they also have some disadvantages. The positive aspects include increased protein stability and activity, increased cellular uptake and protection against degradation. On the other hand, the negative points include the possibility of modifying the structure of the protein, which can lead to the loss of its biological activities, and an abnormal unfolding that can cause an unwanted immune response [37].

To overcome these limitations, we sought to encapsulate bovine Lf (bLf) in stimuli-responsive therapeutic nanoformulations, in order not only to preserve its biological activity but also to promote its targeting and controlled release [27,28]. For this purpose, gold and manganese ferrite NPs were prepared and incorporated in the membrane of liposomes and in the aqueous core of liposomes, respectively, forming aqueous plasmonic magnetoliposomes (PMLs). Two lipid formulations were tested, a natural phospholipid mixture (egg lecithin, Egg-PC) and 100% dipalmitoylphosphatidylcholine (DPPC), whose transition temperature is near that used in mild magnetic hyperthermia treatments. bLf was incorporated into these PMLs, and both the encapsulation efficiency and its release profile were determined. Finally, the antifungal activity of bLf-loaded PMLs, as well as their uptake mechanism, were assessed using *Saccharomyces cerevisiae* by counting the colony forming units and using fluorescence microscopy, respectively. The results are promising for the future use of these bLf-loaded multifunctional liposomes in antifungal therapeutic applications.

## 2. Materials and Methods

### 2.1. Preparation of Manganese Ferrite Nanoparticles

Manganese ferrite magnetic nanoparticles were prepared using the citrate stabilization method, adapted from a protocol previously described [38]. Therefore, to 19 mL of ultrapure water at 100 °C, 294.1 mg of trisodium citrate dihydrate (1.0 × 10^−3^ mol) and 1058 µL of sodium hydroxide solution (19.9 × 10^−3^ mol) were added. Then, 359.5 mg of iron chloride hexahydrate (1.33 × 10^−3^ mol) and 111.5 mg of manganese sulfate monohydrate (6.6 × 10^−4^ mol) were dissolved in 1 mL of ultrapure water and added drop by drop to the above mixture under stirring. In this step, the colorless solution changed to a dark color. After 2 h, the solution was cooled to room temperature and washed with water and ethanol, in a 1:1 ratio, via magnetic decantation. Finally, the NPs were dried and stored in a closed container. A stock solution (20 mM) was prepared with 23.2 mg of magnetic nanoparticles in 5 mL of ultrapure water.

### 2.2. Preparation of Gold Nanoparticles

Gold nanoparticles were synthesized using the citrate reduction method described in [39] to obtain spherical Au NPs. For that, 17 µL of chloroauric acid (1.6 × 10^−3^ M) and 7.35 mg of trisodium citrate dihydrate (5.0 × 10^−4^ M) were added to a conical flask, containing 50 mL of ultrapure water, with stirring. To 1.5 mL of ultrapure water, 5.67 mg of sodium borohydride (0.1 M) was added and placed in the refrigerator for a few minutes. Subsequently, this solution was added, drop by drop, to the flask, under magnetic stirring. At this point, there was a change in color to red ruby. Stirring was continued for one hour and, at the end of the process, the gold nanoparticles were stored in the refrigerator.

#### Functionalization with 11-Mercaptoundecanoic Acid and Octadecanethiol

After preparing the gold NPs, they were functionalized through a ligand exchange process. For this purpose, two different ligands were chosen, 11-mercaptoundecanoic acid (MUA) and octadecanethiol (ODT).

In the case of MUA, 1.42 mg of MUA (1.3 × 10^−3^ M) was added in 5 mL of ultrapure water with 2.6 µL of sodium hydroxide solution (0.01 M) and added to 5 mL of the AuNPs in water. The solution was sonicated for 1 h and then incubated for 24 h in a procedure adapted from [39]. The next day, 20 µL of a magnesium chloride solution (0.1 M) was added to promote the precipitation of nanoparticles, and three washes were performed with centrifugation (8500 rpm) for 10 min to eliminate the excess MUA. The nanoparticles (AuNPs_MUA) were then resuspended in 5 mL of ultrapure water.

For the covering with ODT, a solution was prepared with 5 mL of ethanol and 2.86 mg of ODT (1.0 × 10^−3^ M) and added to 5 mL of AuNPs in water. The mixture was sonicated for 15 min and incubated for 2 h at room temperature. Then, to eliminate the ODT that did not bind to gold, three washing steps were performed through centrifugation (8500 rpm) for 10 min with ethanol and, at the end, it was resuspended in 5 mL of chloroform. This solution (AuNPs_ODT) was stored in the refrigerator.

### 2.3. Preparation of Plasmonic Magnetoliposomes (PMLs)

Plasmonic magnetoliposomes were synthesized using the ethanolic injection method, adapted from [40]. The magnetic NPs were incorporated in the aqueous core and the gold ones in the membrane. Two different formulations were prepared, one with the lipid mixture Egg-PC and another with DPPC. Additionally, magnetoliposomes were prepared with either AuNPs_MUA or with AuNPs_ODT.

To an Eppendorf tube, the lipids Egg-PC or DPPC (1.0 × 10^−3^ M) and AuNPs_MUA or AuNPs_ODT (3.2 × 10^−4^ M) were added, and an ultrapure nitrogen stream was used to evaporate the solvent quickly. At this point, 300 µL of ethanol (or diethyl ether) and the NBD-labelled lipid NBD-C_12_-HPC (1.0 × 10^−6^ M) were added. This solution was added, drop by drop, to a glass tube with 5 mL of ultrapure water with MnFe_2_O_4_ NPs solution (1.0 × 10^−4^ M) under stirring.

### 2.4. Bovine Lactoferrin Encapsulation

bLf was encapsulated in PMLs along with the nanoparticles. The protein was acquired from DMV (Veghel, The Netherlands), with a purity of about 80%, 3.5% moisture and 21% iron-saturated, according to the manufacturer. In this procedure, 187 µL of a stock solution of bLf in water (3.9 mM) was placed in the aqueous part (3 mL), together with 15 µL of a stock solution of magnetic nanoparticles in water (20 mM). The lipid solution, composed of 150 µL of a stock solution of Egg-PC in chloroform (20 mM) and 600 µL of a solution of AuNPs_MUA in water (1.6 mM), was evaporated using ultrapure nitrogen, redissolved in 300 µL of ethanol and injected into the aqueous part, forming bLf-loaded PMLs.

### 2.5. Preparation of Small Unilamellar Vesicles (SUVs) as Membrane Models

As membrane models, small unilamellar vesicles were used. The preparation was accomplished using the ethanolic injection method and started by drying 350 µL of a soybean lecithin solution (20 mM) to a final concentration of 0.001 M under an ultrapure nitrogen stream to form a thin film. Then, 300 µL of ethanol and, in some cases, 64.5 µL of a solution of Nile Red 1.1 × 10^−4^ M in ethanol to obtain a final concentration of 1.0 × 10^−6^ M were added. The above solution was added, drop by drop, to 7 mL of ultrapure water under stirring.

### 2.6. Characterization of Nanoparticles and Magnetoliposomes

#### 2.6.1. UV–Vis–NIR (Ultraviolet–Visible–Near-Infrared) Absorption

Absorption spectra were determined on a Shimadzu UV–Vis–NIR spectrophotometer, model UV-3600 Plus (Shimadzu Corporation, Kyoto, Japan). The spectrum of the magnetic nanoparticles dispersed in water was measured in a wavelength range from 220 nm to 820 nm, with a concentration of 4.0 × 10^−4^ M, and for the AuNPs and AuNPs_MUA dispersed in water and AuNPs_ODT dispersed in chloroform, between 250 nm and 1300 nm. The concentration used for AuNPs dispersions was 3.2 × 10^−4^ M.

#### 2.6.2. X-ray Diffraction

X-ray diffraction (XRD) analyses of MnFe_2_O_4_ were performed using a PAN’alytical X’Pert PRO diffractometer (Malvern Panalytical Ltd., Malvern, UK), operating with CuK_α_ radiation, in Bragg–Brentano configuration, from the University of Trás-os-Montes and Alto Douro (UTAD), Vila Real, Portugal.

#### 2.6.3. Fourier-Transform Infrared Spectroscopy (FTIR)

A PerkinElmer Spectrum Two^TM^ IR spectrometer (PerkinElmer Inc., Waltham, MA, USA) with a diamond ATR (Attenuated Total Reflection) single reflection accessory was used. PerkinElmer Spectrum 10 Software (PerkinElmer Inc., Waltham, MA, USA) was used to draw the spectra. The analysis was performed in the mid-infrared wavenumber range, at a resolution of 4 cm^−1^, averaging 4 scans.

#### 2.6.4. Magnetic Properties and Hyperthermia

Magnetic measurements were performed in a Superconducting Quantum Interference Device (SQUID) magnetometer, Quantum Design’s MPMS 3 model (Quantum Design Inc., San Diego, CA, USA), from the Institute of Materials Physics of the University of Porto.

Magnetic hyperthermia assays were performed using a home-built hyperthermia setup to obtain the heating profile of the magnetic nanoparticles under AFM. For these measurements, the field frequency was fixed at 155 kHz under a magnetic field of 11 mT. The value of SAR was calculated in a program using IAM (Incremental Analysis Method). The heating was monitored for 30 min under the influence of the AFM, and the cooling was recorded over 30 min after AFM shutdown.

#### 2.6.5. Photothermia Assays

Photothermia tests were performed to evaluate the heating capacity of the nanoparticles and plasmonic magnetoliposomes. This study was carried out using an experimental setup assembled in the laboratory, consisting of a sample holder, a continuous light laser (Thorlabs Inc., Newton, NJ, USA) of 532 nm wavelength and 200 mW of power, which irradiated the sample placed in a glass cuvette (10 mm), and a K-type thermocouple connected to a digital multimeter Agilent U1242A (Agilent Technologies, Santa Clara, CA, USA) was used for temperature monitoring. Each assay was irradiated for 30 min (heating), followed by 30 min with cooling (laser off), and the temperature was recorded over time. Another thermocouple was kept in a control cuvette to account for changes in room temperature.

#### 2.6.6. Dinamic Light Scattering and Electrophoretic Light Scattering

The average hydrodynamic size and the zeta potential of the manganese ferrite and gold nanoparticles, as well as the magnetoliposomes, were measured using an Anton Paar equipment, Litesizer 500 model (Anton-Paar GmbH, Graz, Austria). Polystyrene or quartz cuvettes (as needed) of 10 mm of optical path and a Univette cuvette were used for the measurements. The concentrations of the solutions of AuNPs were 1.6 mM, for magnetic nanoparticles were 2.0 × 10^−4^ M and for magnetoliposomes 1.3 × 10^−3^ M. A study of the size and zeta potential of NPs as a function of pH was carried out using PBS (phosphate-buffered saline) buffer solutions.

#### 2.6.7. Transmission Electron Microscopy

Images of the magnetic nanoparticles were obtained through transmission electron microscopy (JEOL JEM1010 (100 kV)) at the Center for Scientific and Technological Research Support (CACTI) of the University of Vigo, Spain. The samples were subjected to ultrasonication and then deposited on copper grids with carbon and Formvar.

TEM images were processed using ImageJ software (version 1.53t, National Institutes of Health (NIH), Bethesda, MD, USA), and the resulting histograms were fitted to a Gaussian distribution.

#### 2.6.8. Fluorescence Spectroscopy Measurements

Fluorescence measurements were recorded using the Fluorolog-3, model FL-1039 (HORIBA Jobin Yvon IBH Ltd., Glasgow, UK), equipped with Glan-Thompson polarizers, double monochromators in excitation and emission and a cuvette holder with temperature control.

##### Fluorescence Emission Studies

To verify the incorporation of the nanoparticles in liposomes, assays based on fluorescence emission (quenching measurements) were performed, using NBD-C_12_-HPC as a fluorescent-labelled lipid, with λ_exc_ = 450 nm. In this labelled lipid, the nitrobenzoxadiazole (NBD) probe was bound in the acyl chains of phospholipids and had good photophysical properties, such as high fluorescence quantum yield, good sensitivity to the environment and possible adaptation, either as a donor or acceptor, for FRET (Förster Resonance Energy Transfer) assays [41].

##### Fusion Assays with Membrane Models

The interaction of the plasmonic magnetoliposomes with membrane models (SUVs) was determined via FRET, using the fluorescent-labelled lipid NBD-C_12_-HPC (0.001 mM), which acted as the energy donor, and the Nile Red dye (0.001 mM) as the acceptor. The NBD-labelled lipid was incorporated into the PMLs and the Nile Red dye into the SUVs. The Nile Red probe is an uncharged and hydrophobic molecule, and its fluorescence depends on the polarity of the environment where it is located. In polar media, it presents a redshift at maximum emission, along with fluorescence quenching [42].

##### Anisotropy Measurements

Fluorescence anisotropy measurements were also performed to study the phase transition temperature of DPPC liposomes and different concentrations of plasmonic nanoparticles in the membrane. The lipid membrane probe 1,6-diphenyl-1,3,5-hexatriene (DPH) was incorporated into the liposomes, and its fluorescence anisotropy was measured as function of temperature, using polarizers in vertical and horizontal position, in excitation and emission, for temperatures below and above the lipid transition temperature, T_m_ (from 25 °C to 55 °C).

The fluorescence anisotropy, *r*, is experimentally determined (as the average of several experimental points) using Equation (1):

(1) $$r=\frac{I_{VV}-{GI}_{VH}}{I_{VV}+{2GI}_{VH}}$$

where I_VV_ and I_VH_ are the intensities of the emission spectra obtained with vertical and horizontal polarization, respectively (for vertically polarized excitation light), I_HV_ and I_HH_ are the emission intensities obtained with vertical and horizontal polarization (for horizontally polarized excitation light) and $G=I_{HV}/I_{HH}$ is the instrumental correction factor.

#### 2.6.9. Encapsulation Efficiency of Bovine Lactoferrin

The encapsulation efficiency (EE%) of bLf in PMLs was determined by measuring the fluorescence emission of the compound itself using Amicon filters. bLf solutions were prepared (in water) with known decreasing concentrations to obtain a calibration curve. The fluorescence emission spectrum was plotted, and the percentage of compound present was calculated, taking into account the calibration curve obtained above, using Equation (2) [43]:

(2) $$\mathrm{EE}(\%)=\frac{{\left[bLf\right]}_{t}-{\left[bLf\right]}_{non\text{-}encapsulated}}{{\left[bLf\right]}_{t}}\times 100$$

where [bLf]_t_ is the total concentration of bLf that was added to magnetoliposomes preparation and [bLf]_non-encapsulated_ is the concentration of bLf that was not incorporated in the nanosystems.

#### 2.6.10. Release Assays

To evaluate the bLf release profile from PMLs, assays were performed using rich medium, Yeast Extract Peptone Dextrose (YEPD) medium, and Tris-HCl buffer at 30 °C and 37 °C.

YEPD medium was prepared with 250 mL of deionized water, 1% (*w*/*v*) yeast extract, 2% (*w*/*v*) bactopeptone and 2% (*w*/*v*) glucose. For the buffer, to 50 mL of deionized water, 60.57 mg of Trizma^®^ base was added to obtain a final concentration of 0.01 M, and the pH was adjusted with HCl to pH = 7.2.

Hereupon, the non-encapsulated part in the Amicon filters was removed, and the YEPD medium was placed in one tube and the Tris-HCl Buffer in the other. The two tubes were placed in a 30 °C incubator with agitation (200 rpm), and samples were taken over three consecutive days. The process was repeated for the assay at 37 °C, which was placed in an incubator at 150 rpm. Two calibration curves were performed, with different known concentrations of bLf in YEPD medium and Tris-HCl buffer.

### 2.7. Biological Assays

#### 2.7.1. Growth Conditions

*S. cerevisiae* wild-type (wt) strain BY4741 cells were grown on rich YEPD medium. Growth was performed overnight at 30 °C and 200 rpm until attainment of the exponential phase (OD640 of 0.7).

#### 2.7.2. In Vitro Cytotoxicity Assays

The cytotoxicity of bLf-loaded PMLs was compared with unloaded PMLs, as well as with free bLf, by counting the colony forming units (CFUs). The treatment with bLf and PMLs was performed as previously described for bLf alone [44]. Briefly, after cells reached the exponential phase, they were harvested to a final OD640 of 0.5 and resuspended in 10 mM Tris-HCl buffer pH 7.2. Afterwards, cells were incubated at 30 °C for up to 90 min with free bLf, unloaded PMLs and bLf-loaded PMLs at different concentrations (62.5, 125 and 250 μg/mL), using stock solutions of 4 mM for bLf and of 10 mg/mL for PMLs and PMLs + bLf. At each timepoint analyzed, serial dilutions were performed for each condition, and then 5 drops of 40 μL of the last dilution (10^−4^) were dropped into Petri dishes containing YEPD plus 2% (*w*/*v*) agar, and the OD640 was read. Petri dishes were incubated at 30 °C for 48 h, and the grown colonies were counted.

The same method was applied to compare the cell viability of the endocytosis-defective mutant strain (*end3*Δ) [45] with the wt strain upon treatment with unloaded PMLs and bLf-loaded PMLs. In this assay, only the highest concentration (250 μg/mL) was tested for different timepoints (15, 30 and 90 min).

#### 2.7.3. Nanosystem Internalization Assays

The *S. cerevisiae* wt strain BY4741 was used to evaluate the internalization of PMLs, unloaded or loaded with bLf, using a methodology adapted from [46]. Cells were resuspended in 10 mM Tris-HCl buffer pH 7.2 and incubated with DPH (7.5 µM) labelled PMLs unloaded or loaded with bLf (250 µg/mL) for 15, 30, 60 and 90 min, at 30 °C and 200 rpm. Samples were centrifuged for 5 min at 3000× *g* and resuspended in 500 µL of PBS 1× (0.137 M NaCl, 2.7 mM KCl, 10 mM Na_2_HPO_4_, 1.8 mM KH_2_PO_4_, pH 7.4). Finally, further centrifugation (3000× *g*, 2 min) was performed, and 450 µL of the obtained supernatant was removed, and the cells were resuspended in the remaining 50 µL. Samples were observed under a fluorescence microscope (Leica Microsystems DM-5000B, Wetzlar, Germany) with a 100× oil immersion objective and with the appropriate filter settings for DPH. The images were obtained with a Leica DCF350FX digital camera (Leica Camera AG, Wetzlar, Germany) and processed with LAS (Leica Microsystems Software; https://www.leica-microsystems.com; accessed on January 2023).

#### 2.7.4. Nanosystem Cellular Uptake under Inhibitory Conditions

Nanosystem uptake experiments in wt cells were also performed using three different inhibitory conditions, namely: incubation with the endocytosis inhibitor methyl-β-cyclodextrin (MβCD, 5 mg/mL) or with the glycolysis inhibitor 2-deoxyglucose (2-DG, 20 nM) and, also, incubation at 4 °C to inhibit cellular metabolism [44,46]. These experiments were carried out using the same methodology as in 2.7.2, modifying only one step. In this case, after changing the medium to Tris-HCl buffer, the cells were incubated under different inhibitory conditions for 30 min. For the inhibitory condition at 4 °C, the temperature was maintained throughout the entire process. After the 30 min incubation under the different inhibitory conditions, DPH (7.5 µM)-labelled bLf-loaded PMLs (250 µg/mL) were added and incubated for 15, 30, 60 and 90 min with yeast cells, treated with the respective inhibitor and then observed under the fluorescence microscope.

## 3. Results and Discussion

### 3.1. Nanoparticles Characterization

#### 3.1.1. UV–VIS–NIR Absorption Spectroscopy

**Figure 1:**
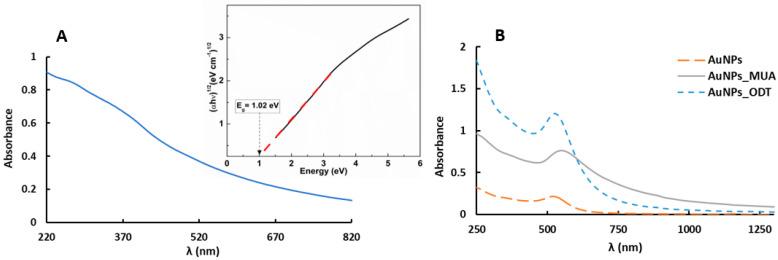
(**A**) Absorption spectrum of MnFe_2_O_4_ NPs dispersed in water (4.0 × 10^−4^ M) synthesized by the citrate stabilization method. Inset: Tauc plot of manganese ferrite nanoparticles. (**B**) Absorption spectrum of AuNPs and AuNPs_MUA dispersed in water (3.2 × 10^−4^ M) and AuNPs_ODT dispersed in chloroform (3.2 × 10^−4^ M), synthesized by the citrate reduction method.

The absorption spectrum of magnetic nanoparticles of manganese ferrite is shown in Figure 1. This type of ferrite was chosen for its higher magnetic susceptibility when compared to ferrites composed of other transition metals [47]. Figure 1A shows a characteristic spectrum of these nanoparticles [40], with notable absorption in the UV/Visible region. An important parameter to evaluate the optical characteristics of the nanoparticles is the band gap. Equation (3) allows us to determine the band gap energy (*E_g_*) by creating a Tauc plot:

(*αhν*)^*n*^ ∝ (*hν* − *E*_*g*_) (3)

where *α* is the absorption coefficient, *h* the Planck constant, *ν* the frequency of light and *n* is related to the nature of the transition, being equal to 2 for a direct semiconductor and 1/2 for an indirect one.

For the synthesized manganese ferrite nanoparticles, the Tauc plot shown in Figure 1A (inset) allows us to confirm an indirect semiconductor material with a band gap energy value of 1.02 eV, which is in agreement with the value of 0.98 eV reported by Rafique et al. [48]. Regarding the gold nanoparticles, in their absorption spectra, represented in Figure 1B, the presence of a characteristic and well-defined plasmonic band is verified. For the neat Au nanoparticles (AuNPs) in water, the maximum peak at 520 nm points to small nanoparticles of around 5–10 nm size [49]. This small size is relevant for the proposed application, as we aimed to prepare AuNPs functionalized with suitable ligands for anchoring them in the lipid membrane but without significantly perturbing the membrane behavior. AuNPs_MUA (NPs covered with 11-mercaptoundecanoic acid) in aqueous media exhibit a plasmonic band at 548 nm, in accordance with the results reported by Ansar et al. [39]. For AuNPs_ODT (gold NPs covered with octadecanethiol in chloroform), the plasmon band is centered at 525 nm, which is also in agreement with previous reports for hydrophobic gold nanoparticles [50]. The position of the plasmonic band must be taken into account in the assessment of the photothermal capabilities of AuNPs.

#### 3.1.2. X-ray Diffraction Analysis of MnFe_2_O_4_ NPs

**Figure 2:**
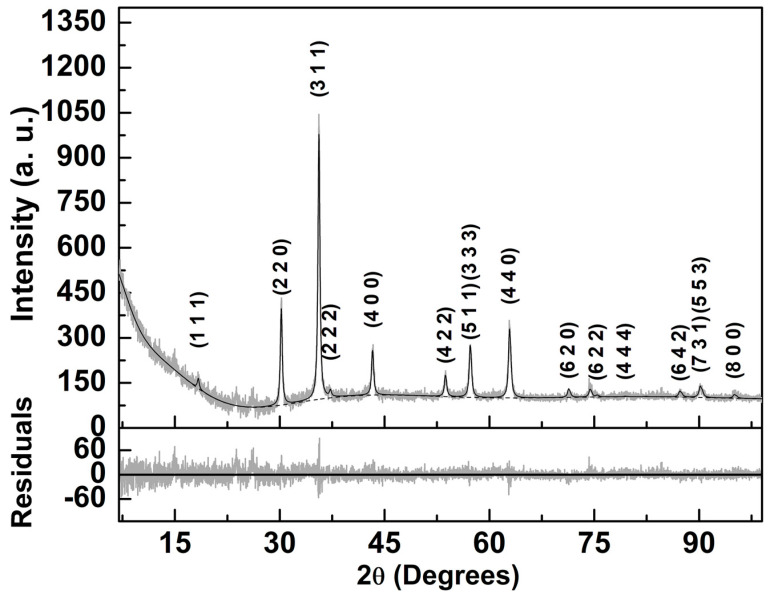
X-ray diffraction pattern of manganese ferrite nanoparticles and corresponding Rietveld analysis with Miller indices.

X-ray diffraction analysis is essential to obtain the crystallinity and degree of purity of the synthesized magnetic NPs. An XRD diffractogram is shown in Figure 2, and it was analyzed with Profex software (version 4.3.6) [51], using a Rietveld analysis implemented by BGMN [52], starting from the CIF file number 1010131 (space group Fd-3m:1) and resulting in a fit with χ^2^ = 1.00 and R_P_ = 6.2. The lattice parameter of 8.356 Å was slightly lower than the one included in the CIF file (8.515 Å).

The XRD pattern proves the synthesis of magnetic NPs (100% MnFe_2_O_4_) with a crystalline structure and 28 nm size (Figure 2). The diffraction peaks of the MnFe_2_O_4_ NPs are observed at 2θ = 18.3° (1 1 1), 30.2° (2 2 0), 35.6° (3 1 1), 37.2° (2 2 2), 43.2° (4 0 0), 53.7° (4 2 2), 57.2° (5 1 1) (3 3 3), 62.8° (4 4 0), 71.3° (6 2 0), 75.4° (6 2 2), 79.4° (4 4 4), 87.2° (6 4 2), 90.1° (7 3 1) (5 5 3) and 95.0° (8 0 0). The relative intensities are compatible with a full inverted structure with preferred orientation effects taken into account, considering the crystal symmetry, as implemented in BGMN model [52].

#### 3.1.3. Magnetic and Structural Properties of MnFe_2_O_4_ Nanoparticles

**Figure 3:**
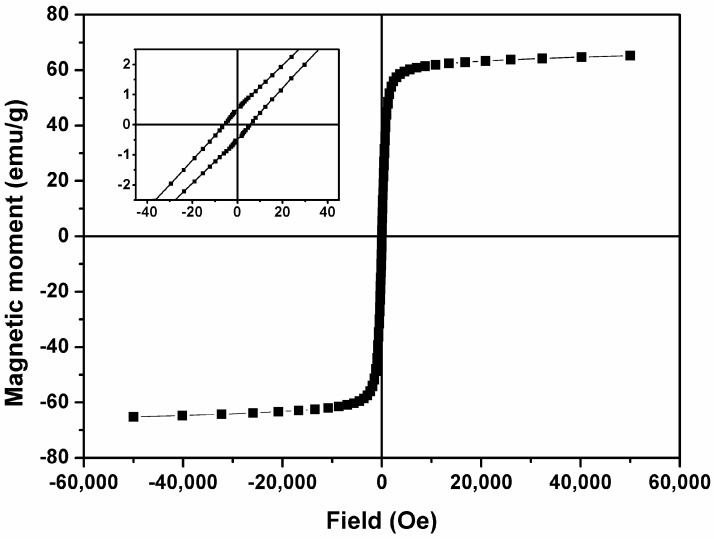
Magnetization hysteresis loop of MnFe_2_O_4_ NPs measured at room temperature. Inset: enlargement of the loop in the low-field region.

The magnetization curve (hysteresis cycle) of the manganese ferrite nanoparticles is shown in Figure 3, and the values of saturation magnetization (M_s_), remanent magnetization (M_r_), coercive field (H_c_) and the ratio between M_r_/M_s_ are summarized in Table 1.

The saturation magnetization value, 65.32 emu/g, is significantly larger than the ones previously reported for the same type of nanoparticles [38,40], evidencing an optimized synthesis method. The ratio between the remanent magnetization and the saturation magnetization, M_r_/M_s_ (magnetic squareness value), points to a nanomaterial with superparamagnetic behavior, characterized by M_r_/M_s_ lower than 0.1 (Table 1), indicating that 90% of the magnetization is lost after removing the applied magnetic field. This behavior of the nanoparticles is crucial to decrease the toxicity and, therefore, points to a potential use in biomedicine.

**Figure 4:**
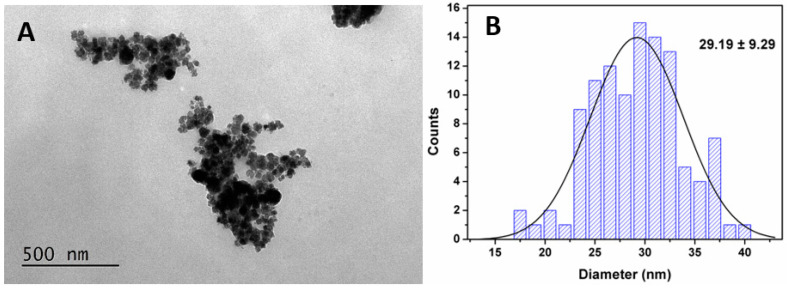
(**A**) TEM image of manganese ferrite nanoparticles and (**B**) size histogram of (**A**) and fitting to a Gaussian distribution.

To obtain the shape and size distribution of the synthesized MnFe_2_O_4_ nanoparticles, they were subjected to characterization via electron microscopy. TEM images reveal that the magnetic nanoparticles have roughly a spherical shape, with the presence of some aggregation (Figure 4A), which may be due to drying during sample preparation.

The TEM image of the nanoparticles was treated with ImageJ software (version 1.53t), and about 100 particles in the image were considered for the determination of the size histogram (Figure 4B). A size distribution of 29.19 ± 9.29 nm was obtained from the histogram shown in Figure 4B. These results are in very good agreement with the ones obtained from XRD.

#### 3.1.4. FTIR and Structural Characterization of Gold Nanoparticles

**Figure 5:**
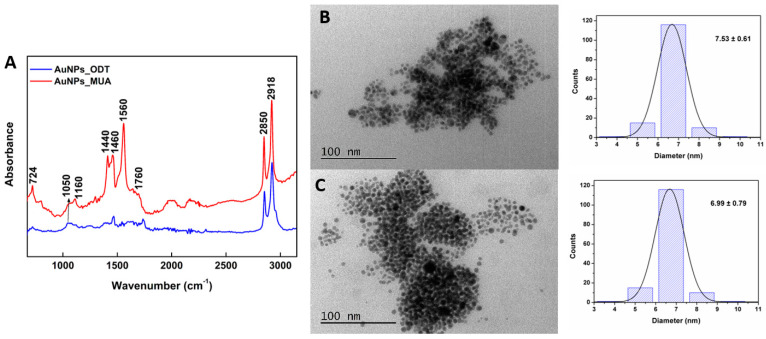
(**A**) FTIR spectra of AuNPs functionalized with ODT and with MUA. (**B**) TEM images of AuNPs_MUA and corresponding size histogram. (**C**) TEM images of AuNPs_ODT and corresponding size histogram.

FTIR measurements were performed to confirm the functionalization of the gold nanoparticles, and the corresponding infrared spectra are shown in Figure 5A.

The peaks at 2850 and 2918 cm^−1^ are assigned to the symmetric (ν_s_) and asymmetric (ν_a_) C–H stretching vibration of the CH_2_ groups, respectively. The peak at 724 cm^−1^ accounts for C–H bending (stronger peak in AuNPs_MUA), and the one around 1050 cm^−1^ accounts for C–C stretching. For the Au nanoparticles functionalized with MUA, it is also possible to detect the carboxylic acid signature in the FTIR spectrum, namely the peaks around 1440 and 1460 cm^−1^ (O–H bending modes), a shoulder around 1760 cm^−1^ (C=O stretching), as well as a peak in the region 1160–1210 cm^−1^ (C–O stretching). In both samples, the absence of a peak at 2550–2600 cm^−1^, due to S–H stretching of the free thiol group, indicates the successful interaction of sulfur to gold nanoparticles, with the formation of S–Au bonds [53,54].

TEM images (Figure 5B,C) reveal that gold nanoparticles are very small (as expected from the LSPR peak obtained at 520 nm), presenting a uniform spherical shape. Size distributions of 7.53 ± 0.61 nm and 6.99 ± 0.79 nm were obtained for the AuNPs_MUA and AuNPs_ODT, respectively, in accordance with the size between 5 and 10 nm estimated from the absorbance peak [49].

DLS measurements of gold nanoparticles in aqueous media show that the highly hydrophobic gold nanoparticles (covered with ODT) and the amphiphilic ones (covered by MUA) are aggregated in solution (especially the ones with ODT) (Table 2). The strong decrease in the zeta potential absolute value for AuNPs_ODT is a result of the surface coverage with a neutral molecule, while MUA exhibits a negative carboxylate group.

#### 3.1.5. Specific Absorption Rate (SAR)

**Figure 6:**
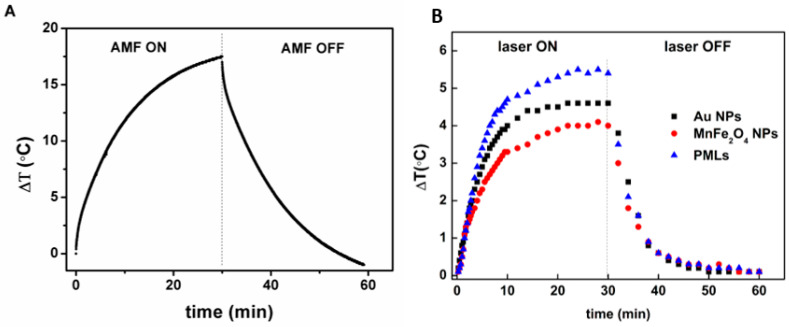
(**A**) Heating and cooling curve of an aqueous dispersion of MnFe_2_O_4_ NPs as a function of time, under AMF (11 mT and 155 kHz). (**B**) Heating and cooling curve of aqueous dispersions of AuNPs_MUA, MnFe_2_O_4_ NPs and plasmonic magnetoliposomes (PMLs) irradiated with a 532 nm continuous light laser (0.2 W/cm^2^).

For the assessment of magnetic hyperthermia capabilities, a stable aqueous dispersion of magnetic nanoparticles (35 mg/mL) was subjected to an alternating magnetic field of 11 mT and a frequency of 155 kHz, as previously mentioned. The heating profile is displayed in Figure 6A. The potential of the nanoparticles as photothermia agents was also evaluated for manganese ferrite NPs, for Au NPs covered with MUA (AuNPs_MUA) and for plasmonic magnetoliposomes (PMLs). For that, the heating curves of aqueous dispersions (1 mg/mL) were measured under irradiation of a continuous laser of 532 nm (Figure 6B). SAR values were determined according to Equation (4):

(4) $$SAR=C\frac{\Delta T}{\Delta t}\times \frac{m_{s}}{m_{NPs}},$$

where C is the specific heat capacity of the medium (4.186 J g^−1^ K^−1^), ΔT/Δt is the initial slope of the temperature curve as a function of time and $m_{s}$ and $m_{NPs}$ are the mass of solvent and nanoparticles, respectively [55]. Table 3 summarizes the magnetic and photothermal hyperthermia results.

The magnetic hyperthermia heating curve shows a large temperature increase of 17.5 °C in 30 min (Figure 6A), with a corresponding SAR value of 4.18 W/g. When AMF shuts down, the aqueous dispersion returns to room temperature after 24 min. Pradhan et al. reported, for MnFe_2_O_4_ spherical NPs synthesized via a co-precipitation method, an SAR value of 97 W/g, under an AMF of 18.8 mT amplitude and 300 kHz frequency [56]. Taking the much lower AMF amplitude and frequency values used here and the appropriate temperature increase for therapeutic action, the MnFe_2_O_4_ NPs obtained in this work are promising as magnetic hyperthermia agents under more safety AMF operating conditions.

Photothermia experiments demonstrate a temperature increase of ca. 4 °C, 4.6 °C and 5.5 °C for magnetic NPs, AuNPs_MUA and PMLs, respectively, upon 30 min of laser irradiation (Figure 6B). The corresponding SAR values are displayed in Table 3. It must be noted that the photothermal performance of AuNPs_MUA may be reduced due to the NPs covering with 11-mercaptoundecanoic acid, which is a self-assembling amphiphilic molecule (with a hydrophobic aliphatic chain and a hydrophilic head carboxylic group) [57]. This character may cause the self-aggregation of MUA-covered gold nanoparticles in aqueous media, thus affecting the heating profile. Nevertheless, considering an initial temperature of 37 °C (normal body temperature), the plasmonic magnetoliposome dispersion would reach ca. 43 °C in half an hour, making it possible to trigger apoptosis in the cells [58]. Photothermia has already revealed advantages when applied in antifungal therapies [59]. Moreover, this rise in temperature can be even larger through the application of an alternating magnetic field using the magnetic hyperthermia capability of the magnetic nanoparticles.

### 3.2. Characterization of Plasmonic Magnetoliposomes

The magnetic and plasmonic nanoparticles were included in liposomes to obtain plasmonic magnetoliposomes suitable as nanocarriers for the transport and release of bLf.

#### 3.2.1. Fluorescence Quenching by Magnetic and Plasmonic NPs

**Figure 7:**
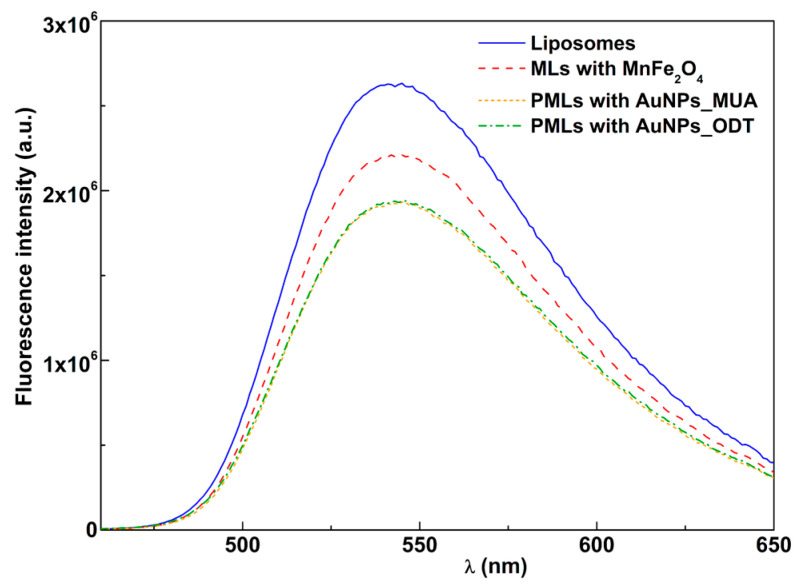
Fluorescence spectra (λ_exc_ = 450 nm) of the labeled lipid NBD-C_12_-HPC (1 × 10^−6^ M) incorporated in Egg-PC liposomes, in magnetoliposomes (MLs) with MnFe_2_O_4_ NPs and PMLs with MnFe_2_O_4_ and AuNPs_MUA or AuNPs_ODT (spectra of both PMLs are almost coincident).

The emission of the fluorescent-labeled lipid NBD-C_12_-HPC (NBD as the fluorophore) included in the lipid formulation in liposomes with only MnFe_2_O_4_ NPs, PMLs with MnFe_2_O_4_ and AuNPs_MUA or AuNPs_ODT and liposomes composed of Egg-PC (the latter without nanoparticles and with the same concentration of lipid) is shown in Figure 7 (similar results were obtained for DPPC-based nanosystems).

In magnetoliposomes, with only MnFe_2_O_4_ NPs, a quenching effect on the NBD fluorescence is verified, which results from the proximity between the dye and the MnFe_2_O_4_ NPs, which absorb in a wide wavelength range. This quenching effect is larger in PMLs with the presence of the two types of nanoparticles. This phenomenon confirms the incorporation of both MnFe_2_O_4_ NPs and AuNPs with MUA or ODT (Figure 7) and can be explained by photoinduced electron transfer and/or an increase in intersystem crossing efficiency due to the heavy atom effect. The last mechanism usually prevails in the emission quenching in this type of nanosystem [60].

#### 3.2.2. Fusion Assays with Membrane Models

**Figure 8:**
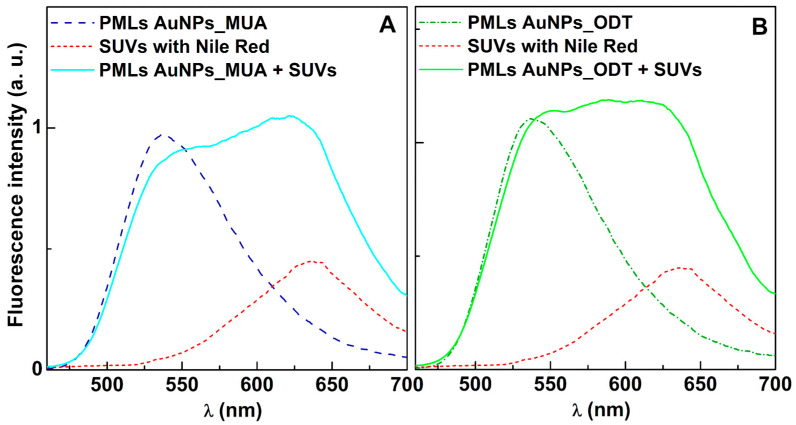
Fluorescence spectra (λ_exc_ = 450 nm) of PMLs of: (**A**) MnFe_2_O_4_ and AuNPs_MUA. (**B**) MnFe_2_O_4_ and AuNPs_ODT, containing the labeled lipid NBD-C_12_-HPC (1 × 10^−6^ M), SUVs containing only Nile Red (1 × 10^−6^ M) and of the mixture of labelled PMLs and SUVs.

To assess the fusion ability of the developed PMLs with models of biological membranes, the labeled lipid NBD-C_12_-HPC was incorporated into the PMLs’ lipid bilayer, while the Nile Red dye was incorporated into small unilamellar vesicles (SUVs) used as membrane models. If fusion between PMLs and SUVs occurs, a FRET process is expected, where the NBD (in PMLs) acts as the energy donor and the hydrophobic dye Nile Red (located in SUVs) acts as the energy acceptor [40]. For resonance energy transfer to occur, the donor–acceptor distance must be lower than 100 Å. Fluorescence spectra of DPPC liposomes containing MnFe_2_O_4_ NPs and AuNPs_MUA (Figure 8A) and MnFe_2_O_4_ and AuNPs_ODT (Figure 8B), before and after interaction with SUVs, were measured by exciting the energy donor (a residual excitation of Nile Red is also detected at this wavelength).

Before the interaction with SUVs, the presence of an emission band was observed in the separate samples of PMLs and SUVs. The band in Figure 8A,B of PMLs has a maximum around 535 nm, corresponding to NBD emission. The band of SUVs has a maximum around 635 nm due to the emission of Nile Red in an aqueous environment [40]. After interaction with the membrane models, there is a slight decrease in the fluorescence band of NBD (donor) and a strong increase in the fluorescence band of Nile Red (acceptor), proving the FRET process. However, due to the significant superposition of NBD and Nile Red emission spectra, the large increase in the latter makes the decrease in NBD fluorescence barely noticeable.

These results are similar to those previously reported for magnetoliposomes containing manganese ferrite nanoparticles [40] and validate the membrane fusion between PMLs and SUVs. Consequently, the results allow us to conclude that PMLs are favorable as drug delivery systems, as they are able to fuse with cell membranes.

#### 3.2.3. Influence of AuNPs in Phase Transition of DPPC

**Figure 9:**
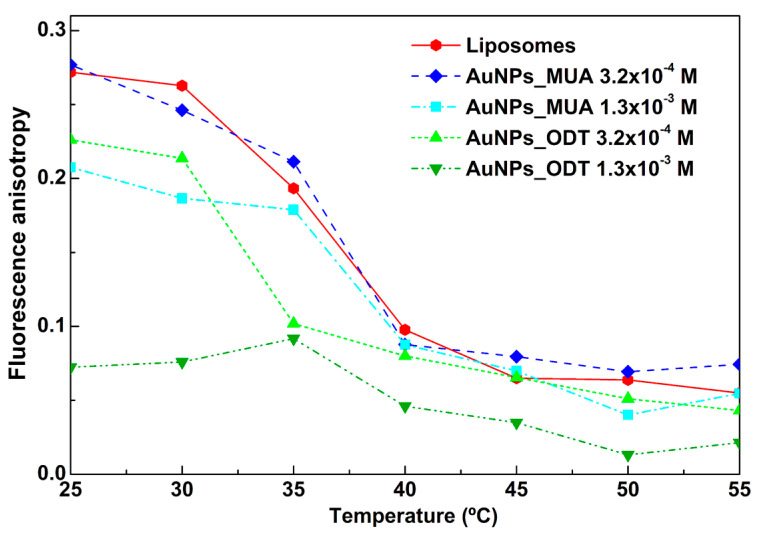
Variation with temperature of fluorescence anisotropy of DPH incorporated into DPPC-based PMLs, with two different concentrations of AuNPs_MUA and AuNPs_ODT, and DPPC liposomes (for comparison).

As previously described, gold nanoparticles were covered with specific ligands to anchor them in the lipid bilayer of PMLs. Therefore, it is important to assess the effect of AuNPs_MUA and AuNPs_ODT, at two different concentrations, on the phase transition of DPPC-based PMLs. For that purpose, the fluorescence anisotropy of DPH, a membrane probe, was measured at varying temperatures. This probe is usually employed to determine changes in membrane fluidity and the transition temperature of phospholipids and has been used for a long time [61,62]. The results are summarized in Figure 9.

It is possible to verify that the incorporation of the AuNPs_ODT into PMLs leads to a notable decrease in fluorescence anisotropy values of the membrane probe DPH. This indicates an increase in the membrane fluidity, with the loss of DPPC phase transition for the higher AuNPs_ODT concentration, pointing to a strong perturbation of membrane dynamics. For the PMLs with AuNPs_MUA, only the higher concentration showed a decrease in DPH anisotropy, indicating that these NPs have a lower influence in membrane fluidity. Moreover, the DPH anisotropy variation is very similar for neat DPPC liposomes and PMLs with AuNPs_MUA at 3.2 × 10^−4^ M, evidencing a transition temperature around 40 °C [63]. For these reasons, the AuNPs_MUA nanoparticles were selected for subsequent biological studies with bLf.

#### 3.2.4. Structural and Surface Charge Characterization

The nanosystem size and polydispersity (PDI) are important parameters that significantly influence, for example, pharmacokinetics, tissue diffusion and kidney excretion [64]. It is generally accepted that the desirable size of drug delivery systems should be between 50 and 200 nm [65]. PDI values reflect the degree of heterogeneity in size distributions, being recognized for this type of application that PDI must be below 0.3, indicating a homogeneous population of the nanocarriers [64]. The hydrodynamic diameter and PDI of the PMLs were measured using DLS (Table 4). All formulations revealed a hydrodynamic diameter around or slightly above 200 nm, except the PMLs with AuNPs_ODT. Generally, the size is narrowly distributed, with a polydispersity index below the 0.3 limit. The presence of the magnetic and plasmonic nanoparticles causes a small increase in size relative to the neat liposomes, which may be due to the gold nanoparticles in the lipid membrane. It was previously shown that the ferrite nanoparticles of transition metals (nickel ferrite, manganese ferrite) enclosed in the aqueous inner volume of magnetoliposomes have very little influence in the nanosystems’ diameter [40,66].

Zeta potential values (Table 4) reveal negatively charged nanosystems for Egg-PC-based liposomes and PMLs, with a slight diminution in the surface charge (being less negative) in the magnetic/plasmonic systems, as expected, since the Au nanoparticles are located at the lipid membrane. For DPPC-based liposomes, an inversion of zeta potential values is detected in the presence of the nanoparticles, seeming to perturb the lipid membrane, exposing the positive choline head group. In general, the zeta potential values higher than ±30 mV point to long-time colloidal stability of the nanosystems [67]. No appreciable variations in both hydrodynamic size and zeta potential are detected after 24 h of preparation, except for DPPC PMLs with AuNPs_ODT. These Au nanoparticles also seem to have a higher influence in the hydrodynamic size of the liposomes when compared with AuNPs_MUA.

**Figure 10:**
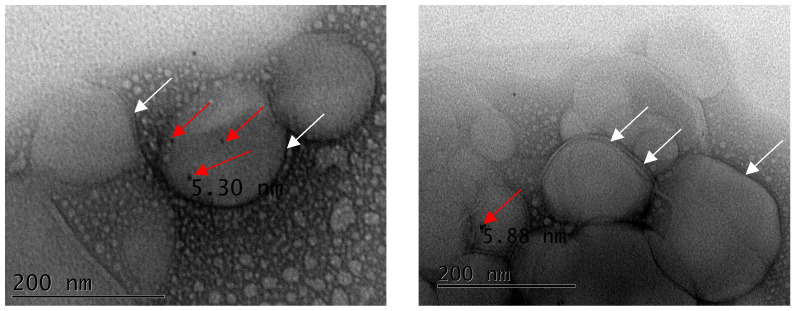
Transmission electron microscopy (TEM) images of Egg-PC PMLs containing AuNPs_MUA (due to the vacuum system of TEM, the structures become aggregated in the grid). The lipid bilayer is marked with white arrows and the AuNPs with red arrows.

TEM images of unloaded PMLs (Figure 10) revealed roughly spherical nanostructures, with sizes around or below 200 nm (in accordance with DLS results, Table 4) and surrounded by a lipid layer. Small Au nanoparticles (the dark spots), with sizes between 5 and 6 nm, are clearly observed at the membrane surface (the MnFe_2_O_4_ NPs are enclosed in the aqueous inner volume of the liposomes). These structures are very similar to the ones reported by Lee and co-workers for vesicles containing gold nanoparticles anchored in the membrane [68].

Taking all the previous characterizations into account, bLf was encapsulated only in Egg-PC plasmonic magnetoliposomes containing manganese ferrite NPs and AuNPs_MUA.

### 3.3. bLf-Loaded Plasmonic Magnetoliposomes

#### 3.3.1. Hydrodynamic Diameter and Zeta Potential

**Figure 11:**
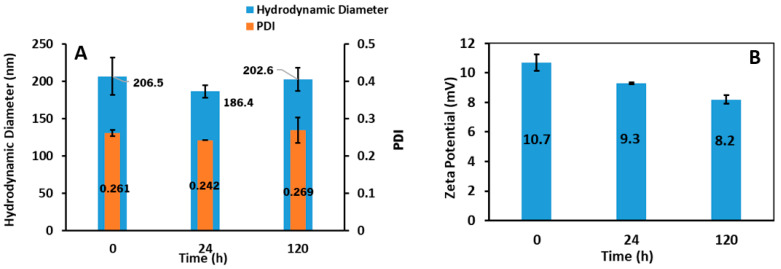
(**A**) Hydrodynamic diameter and polydispersity (PDI) and (**B**) zeta potential of bLf-loaded PMLs at t = 0, 24 and 120 h after preparation.

The hydrodynamic diameter and zeta potential of bLf-loaded PMLs were measured at time 0 (immediately after preparation), at 24 h and 120 h after synthesis to assess their stability over time (Figure 11).

Both size and zeta potential show similar values over time, evidencing the stability of the nanosystems since there are no significant changes after 120 h of their formation. The hydrodynamic diameter of bLf-loaded PMLs is around 200 nm, suitable for biomedical applications. bLf-loaded nanosystems have positive zeta potential values, proving the incorporation of bLf, which is a positively charged molecule [69].

#### 3.3.2. bLf Encapsulation Efficiency and Release Profiles

The encapsulation efficiency, EE(%), of bLf in PMLs was obtained through measurements of fluorescence emission. The maximum fluorescence intensity values of bLf in water (λ_max_ = 300 nm) obtained for the phase corresponding to the non-encapsulated compound allowed for the calculation of the EE(%) according to Equation (2) (Table 5).

The average value of EE percentage obtained (95.6% ± 1.0%) for these nanosystems confirms the incorporation of bLf in the PMLs, as well as their capacity as carriers for this protein. The EE percentage achieved for bLf is significantly higher than the percentage reported for the encapsulation of several proteins in liposomes [70].

The bLf release behavior was studied, over time, to assess the drug release profile from plasmonic magnetoliposomes. Figure 11 shows the percentage of bLf released from PMLs.

Two mathematical models, the Weibull model and first-order kinetic model, were used to better understand the release mechanism of bLf from the PMLs. The Weibull model expresses the fraction of released compounds accumulated (m) in solution at time t, following Equation (5) [71],

(5) $$m=1-{exp}^{-{\left(t-T_{i}\right)}^{\frac{b}{a}}}$$

where *a* denotes the timescale of the process, T_i_ is a location parameter indicating the latency time of the release mechanism and *b* parameter denotes the curve shape. Though the Weibull mathematical model is empirical, a correlation between the model parameters and diffusional mechanism can be established. For *b* > 1, the transport follows a complex release mechanism; *b* ≤ 0.75 indicates Fickian diffusion (in either fractal or Euclidian spaces), and 0.75 < *b* < 1 indicates a combined mechanism between Fickian diffusion and Case II transport.

The first-order mathematical model is described via Equation (6) [72],

(6) $${F\left(\%\right)=M_{0}\times (1-e}^{-kt})$$

where F(%) and M_0_ are the percentage and the total amount of the compound released, respectively, *k* is the first-order rate constant and t is the time.

**Figure 12:**
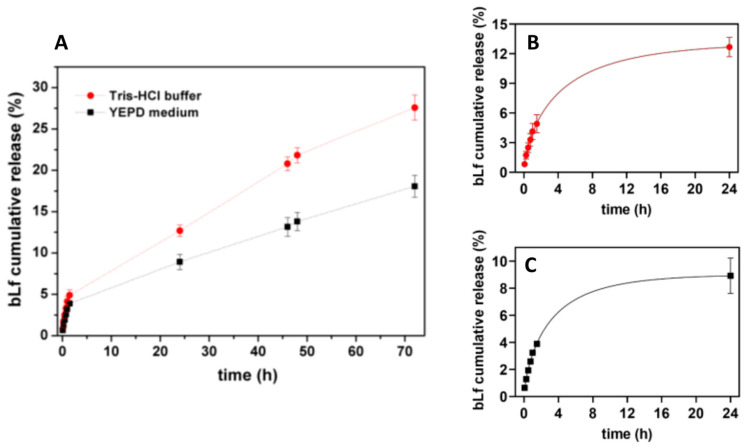
(**A**) bLf release profile from PMLs in Tris-HCl buffer and in YEPD medium for 72 h, at 37 °C. Percentage of bLf released in the first 24 h, fitted to Weibull model: (**B**) in Tris-HCl buffer and (**C**) in YEPD medium.

The release profile of bLf from the plasmonic magnetoliposomes was obtained in both the YEPD medium and Tris-HCl buffer at 37 °C. Figure 12A shows the percentage of bLf cumulative released from PMLs over a 72 h period. The release kinetic data up to 24 h were fitted to Weibull and first-order kinetic models, and the fitting parameters obtained are summarized in Table 6. Figure 12B,C display the experimental data in the first 24 h fitted to the Weibull model (as an example).

The experimental data allow for a good fitting to the Weibull and first-order kinetic models, with high determination coefficients. A slightly better fit was obtained for the Weibull model (Table 5). The parameter *b* indicates a Fickian-type transport in Tris-HCl buffer and a combination of Fickian diffusion and Case II transport mechanism in the YEPD medium. The faster release obtained in the Tris-HCl buffer may be due to a hampered release of bLf in the YEPD medium, considering that this is an amino-acid-rich medium.

### 3.4. Antifungal Activity of bLf-Loaded PMLs

#### 3.4.1. Evaluation of Cytotoxicity and Internalization of bLf-Loaded PMLs

**Figure 13:**
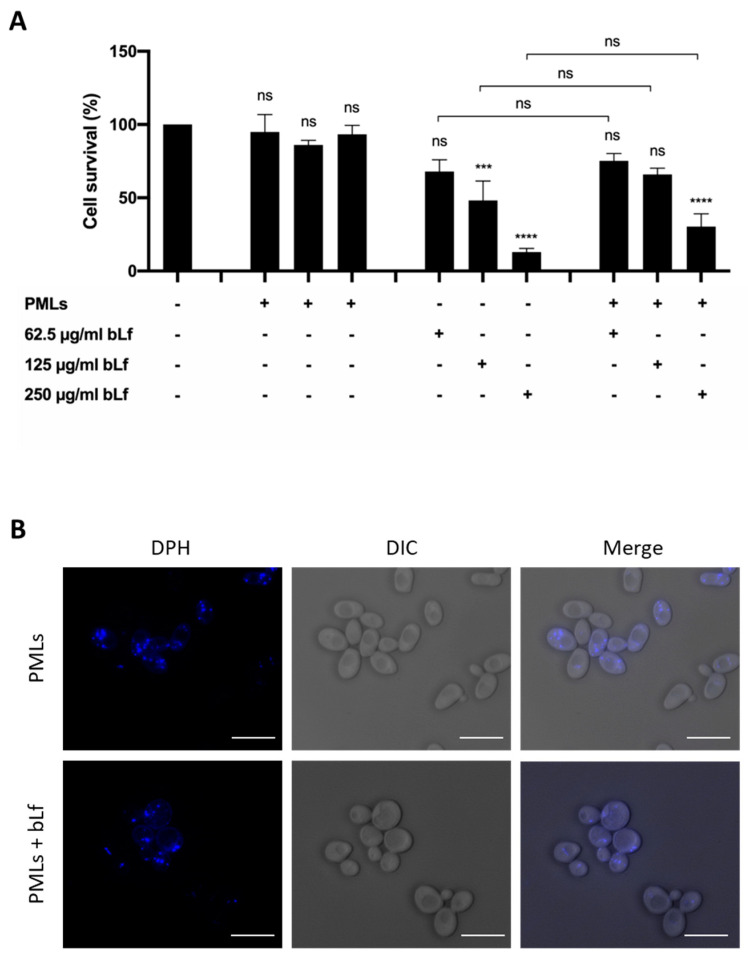
(**A**) Cell viability of *S. cerevisiae* wt strain in Tris-HCl buffer, incubated for 90 min with different concentrations of bLf-unloaded PMLs, bLf-loaded PMLs (PMLs + 62.5, 125 and 250 μg/mL bLf) or of free bLf (62.5, 125 and 250 μg/mL). A control consisting of yeast cells without any treatment is also shown. Cell survival is expressed in percentage, considering 100% the number of CFUs at time 0 for each condition tested. The values presented are the mean and standard derivation of three independent experiments (n = 3). ns—non-significant; ***, **** *p* < 0.001, 0.0001, respectively, in comparison to the untreated control. Significance was evaluated using one-way ANOVA followed by Tukey’s multiple comparison test. (**B**) Fluorescence microscopy images of PMLs and bLf-loaded PMLs (250 µg/mL), labeled with DPH (blue fluorescence), incubated for 90 min at 30 °C with wt cells. Scale bar: 5 μm.

In order to evaluate the antifungal potential of the developed nanosystems, *S. cerevisiae* cells were incubated with bLf-loaded PMLs, as well as with unloaded PMLs and free bLf, and their effect on cell survival was monitored by counting CFUs (Figure 13A).

While unloaded PMLs did not affect cell viability, PMLs loaded with increasing concentrations of bLf caused an increased loss of cell viability, which was not statistically different from that observed for free bLf. Altogether, these results show that PMLs per se are not cytotoxic and are able to transport and release bLf without the loss of antifungal activity.

After confirming that the bLf-loaded PMLs have promising characteristics for antifungal applications, as they preserve the bLf potent yeast killing activity, studies were carried out to confirm their internalization. In these assays, unloaded PMLs and bLf-loaded PMLs were labelled with the fluorescent probe DPH, which is used to visualize hydrophobic regions of membranes in structural and dynamic studies [73]. Through fluorescence microscopy, it was possible to verify that the nanosystems are internalized by yeast cells (Figure 13B).

#### 3.4.2. Study of the Internalization Mechanism of bLf-Loaded PMLs

**Figure 14:**
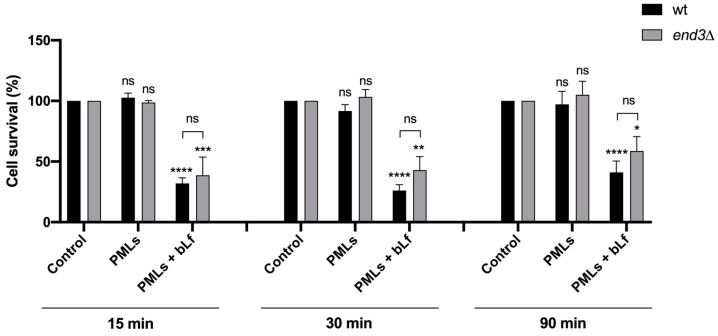
Cell survival of *S. cerevisiae* wt and mutant *end3*Δ cells, in Tris-HCl buffer, incubated for 15, 30 and 90 min with 250 µg/mL of PMLs and bLf-loaded PMLs. A control consisting of yeast cells without any treatment is also shown. Cell survival is expressed in percentage, considering 100% the number of CFUs at time 0 for each condition tested. The values presented are the mean and standard derivation of three independent experiments (n = 3). ns—non-significant; *, **, ***, **** *p* < 0.05, 0.01, 0.001, 0.0001, respectively, in comparison with the control. Significance was evaluated using one-way ANOVA followed by Tukey’s multiple comparison test.

To understand the mechanism of internalization, the *end3*Δ mutant, known to be deficient in endocytosis, was used. This strain lacks the *END3* gene, which encodes a protein known to be involved in endocytosis [44]. Thereunto, the cytotoxicity of unloaded and bLf-loaded PMLs (250 µg/mL) in the wt strain and *end3*Δ mutant was evaluated over time (Figure 14). Although the *end3*Δ mutant tends to show a higher survival than the wt strain, 15 to 90 min after treatment with bLf-loaded PMLs, the differences were not statistically significant (Figure 14).

**Figure 15:**
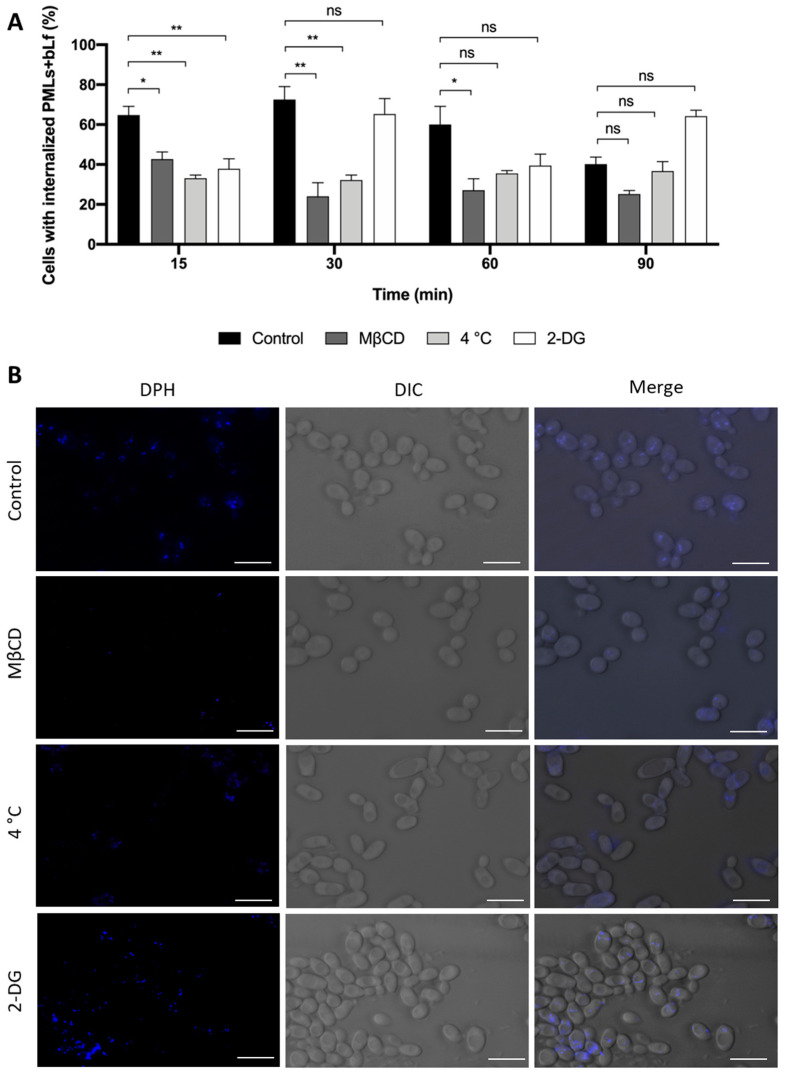
(**A**) Internalization of PMLs + bLf by *S. cerevisiae* wt cells in Tris-HCl buffer after pre-incubation for 30 min with 5 mg/mL MβCD, at 4 °C and with 20 nM 2-DG, followed by incubation for 15, 30, 60 and 90 min with 250 µg/mL of bLf-loaded PMLs. A control consisting of yeast cells without any treatment, at 30 °C is also shown. Internalization is expressed as percentage of cells exhibiting blue fluorescence. The values presented are the mean and standard derivation of three independent experiments (n = 3). ns—non-significant; *, ** *p* < 0.05, 0.01, respectively, in comparison with the control. Significance was evaluated using one-way ANOVA followed by Tukey’s multiple comparison test. (**B**) Fluorescence microscopy images of *S. cerevisiae* cells pre-incubated with MβCD, at 4 °C and with 2-DG for 30 min, and then incubated for additional 30 min with 250 µg/mL of bLf-loaded PMLs labeled with DPH (blue fluorescence). Scale bar: 5 µm.

To further understand the mechanism of internalization of the developed nanocarriers, their uptake was monitored under different inhibitory conditions through fluorescence microscopy, as described above. The analysis of fluorescence microscopy images allowed us to determine the percentage of yeast cells that internalized bLf-loaded PMLs on the untreated control cells and under different inhibitory conditions (Figure 15A). MβCD is a lipid raft disrupting agent known to deplete cholesterol from the plasma membrane. Since it affects membrane fluidity, it inhibits different endocytic pathways, namely fast endophilin-mediated endocytosis, clathrin-independent endocytosis, micropinocytosis and phagocytosis and also caveolin-mediated endocytosis in mammalian cells [73]. 2-DG is a non-metabolizable analogue of glucose that inhibits glycolysis and leads to ATP depletion [44]. In addition, yeast cells were incubated with PMLs at low temperature (4 °C) to ascertain whether diffusion through the plasma membrane may also contribute to PML uptake, as it is temperature dependent [74,75].

Figure 15A demonstrates that 15 min after incubation under the three inhibitory conditions, the internalization of the bLf-loaded PMLs is significantly decreased in comparison with the control, which is maintained over 30 and 60 min for incubation at 4 °C and with MβCD, respectively. Figure 15B allows for a visual observation of the uptake of the nanosystems in each condition. The inhibitory effect on PML internalization induced by pre-treatment with 2-DG indicates that the uptake process depends on the energy generated by glucose metabolism [44], in agreement with the significant reduction in PML uptake at 4 °C [74,75]. However, at longer PML incubation times (60–90 min), both 2-DG and low temperature do not appear to affect PML uptake, which suggests that transmembrane diffusion may also be contributing to PML uptake in yeast cells. This interpretation is reinforced by the observation of a similar uptake of PMLs by MβCD pre-treated and control cells at the 90 min timepoint.

Data from the literature show that micropinocytosis is involved in the uptake of nanosystems with diameters greater than 200 nm [73,76]. As for charged systems, their internalization is known to be mediated by clathrin-mediated endocytosis (CME) and caveolae-mediated endocytosis (CvME) [76,77]. On the other hand, it has been demonstrated that nanoparticles with sizes larger than 8 nm enter cells through energy-dependent processes, generally involving endocytosis [78]. Accordingly, the internalization of the PMLs, herein developed, which have a medium size around 20 nm, is energy- and temperature-dependent, as attested by the results obtained with the 2-DG inhibitor and 4 °C, respectively. In addition, the results with MβCD suggest that an endocytic process is involved in the internalization of PMLs by yeast cells. A similar uptake mechanism was described for resveratrol-loaded liposomes in yeast [46]. Furthermore, it was shown that cationic nanosystems have the ability to bind directly to the anionic head of phospholipids at the cell membrane, which can induce CME internalization [27,28]. As the herein-developed PMLs have a positive charge, their internalization may occur through this specific endocytic pathway. However, further studies are required to better characterize whether a diffusion component, as well as which specific endocytic pathways, is involved in their uptake.

## 4. Conclusions

In this work, a multifunctional nanocarrier containing the bLf protein was developed as a new strategy for bLf-based antifungal applications. Superparamagnetic nanoparticles of manganese ferrite were obtained with sizes around 20 nm and a saturation magnetization of M_s_ = 65.32 emu/g. Furthermore, the assessment of the hyperthermia capability showed strong heating capacity in just 30 min. Plasmonic gold nanoparticles functionalized with 11-mercaptoundecanoic acid (MUA) or octadecanethiol (ODT) were also synthesized. Both types of NPs (magnetic and plasmonic) were encapsulated in liposomes of DPPC and Egg-PC, forming plasmonic magnetoliposomes (PMLs). FRET assays indicated the fusion ability of the developed PMLs with biomembrane models. Fluorescence anisotropy studies of the DPH probe allowed us to conclude that AuNPs_MUA do not significantly alter the membrane fluidity and phase transition temperature of DPPC liposomes, while the same does not happen for AuNPs_ODT. The Egg-PC liposomes were revealed to be more suitable for the incorporation of functionalized AuNPs, being chosen as nanocarriers for bLf and used in the release studies and biological assays. The encapsulation of bLf exhibited an efficiency value of 96 ± 1%, suggesting PMLs as promising nanocarriers for this protein. bLf-loaded PMLs have sizes around 200 nm, are stable for at least 5 days and have a positive zeta potential.

Finally, the biological assays allowed us to demonstrate the PMLs’ promising potential for bLf delivery into yeast cells as they are not cytotoxic per se and are able to transport and release bLf without the loss of its powerful antifungal activity. Moreover, the internalization assays suggest that the uptake of bLf-loaded PMLs is mainly mediated by an energy- and temperature-dependent and MβCD-inhibitable endocytic process. However, the contribution of a diffusion component to PMLs uptake by yeast cells cannot be discarded. All these features constitute an added value of the developed nanosystems for their use as bLf nanocarriers. Indeed, the encapsulation of bLf in the developed nanocarriers may be a solution to preserve its tertiary structure-dependent biological activities and avoid degradation, which will likely increase the local amount of intact bLf reaching the therapeutic target. The biological activity of bLf, in combination with dual hyperthermia (promoted by both magnetic stimuli and laser irradiation), is advantageous for developing therapeutic applications based on bLf, namely in the treatment of antifungal infections.

In summary, the developed multifunctional nanosystems appear as excellent transport vehicles for bLf, allowing for combined magnetic hyperthermia and photothermia and the efficient release of this protein, constituting a promising approach for antifungal therapies.

## Figures and Tables

**Table 1:** Coercive field (H_c_), remanent magnetization (M_r_), saturation magnetization (M_s_) and the ratio between remanent magnetization and saturation magnetization (M_r_/M_s_) for MnFe_2_O_4_ NPs.

	H_c_ (Oe)	M_s_ (emu/g)	M_r_ (emu/g)	M_r_/M_s_
MnFe_2_O_4_ NPs	5.45	65.32	0.52	0.008

**Table 2:** Hydrodynamic size, polydispersity (PDI) and zeta potential values for AuNPs_ODT and AuNPs_MUA compared with the neat AuNPs. SD: standard deviation of three independent measurements.

NPs	Hydrodynamic Diameter ± SD (nm)	PDI ± SD	Zeta Potential ± SD (mV)
AuNPs	84 ± 4	0.27 ± 0.01	−39.9 ± 1.9
AuNPs_MUA	101 ± 39	0.27 ± 0.06	−35.0 ± 1.3
AuNPs_ODT	450 ± 36	0.02 ± 0.01	−6.6 ± 5.7

**Table 3:** Maximum temperature variation (ΔT), slope (ΔT/Δt) and Specific Absorption Rate (SAR) values of MnFe_2_O_4_ (both by magnetic hyperthermia and photothermia), AuNPs_MUA and PMLs.

Nanoparticles	Mechanism	ΔT (°C)	ΔT/Δt (°C/min)	SAR (W/g)
MnFe_2_O_4_	Magnetic hyperthermia	17.5	0.05	4.2
MnFe_2_O_4_	Photothermia	3.99	0.52	2177
AuNPs_MUA	Photothermia	4.58	0.80	3349
PMLs	Photothermia	5.52	0.52	1088

**Table 4:** Hydrodynamic size, polydispersity (PDI) and zeta potential values for DPPC PMLs and Egg-PC PMLs, containing MnFe_2_O_4_ NPs and AuNPs_ODT or AuNPs_MUA, immediately after preparation (0 h) and 24 h after. SD: standard deviation of three independent measurements.

Lipid	System	Hydrodynamic Diameter ± SD (nm)		PDI ± SD		Zeta Potential ± SD (mV)	
		0 h	24 h	0 h	24 h	0 h	24 h
DPPC	Liposomes	186 ± 14	165 ± 25	0.28 ± 0.01	0.30 ± 0.05	−13.2 ± 0.2	−13.6 ± 0.8
	PMLs with AuNPs_MUA	195 ± 7	203 ± 10	0.27 ± 0.04	0.29 ± 0.08	31.1 ± 0.8	30.8 ± 0.4
	PMLs with AuNPs_ODT	261 ± 10	302 ± 55	0.279 ± 0.007	0.248 ± 0.03	28.1 ± 0.8	31.2 ± 0.3
Egg-PC	Liposomes	132 ± 11	140 ± 21	0.24 ± 0.03	0.27 ± 0.01	−27 ± 2	−28 ± 1
	PMLs with AuNPs_MUA	207 ± 18	215 ± 10	0.27 ± 0.04	0.26 ± 0.02	−12.6 ± 0.5	−8.7 ± 0.6
	PMLs with AuNPs_ODT	337 ± 32	334 ± 7	0.28 ± 0.04	0.26 ± 0.02	−16.5 ± 0.4	−14.9 ± 0.2

**Table 5:** bLf encapsulation efficiency (EE%) values in Egg-PC PMLs for three independent measurements, and the respective mean with standard deviation (SD). Systems were prepared with Egg-PC concentration of 1 mM and bLf 0.243 mM.

Assay	EE (%)	Mean ± SD (%)
I	94.7	
II	95.4	95.6 ± 1.0
III	96.6	

**Table 6:** Parameters of Weibull and first-order kinetic models fitted to the bLf release data (in Tris-HCl buffer and YEPD medium) in the first 24 h and respective coefficient of determination (*R*^2^).

	Weibull			First-Order	
	*a*	*b*	*R* ^2^	*k* (min^−1^)	*R* ^2^
Tris-HCl buffer	0.396	0.695	0.999	0.505	0.987
YEPD medium	0.511	0.757	0.999	0.417	0.984

## Data Availability

Not applicable.
